# Quality of Life Among Patients With Epilepsy in Jeddah, Saudi Arabia: A Descriptive Cross‐Sectional Study

**DOI:** 10.1155/nrp/9339047

**Published:** 2026-07-12

**Authors:** Ruba Alharazi, Yara Abushosha, Nouf A. Aldajany, Nouf J. Alreziq, Lujain A. Sallam, Aisha Alhofaian, Loujain S. Sharif

**Affiliations:** ^1^ Medical Surgical Nursing Department, Faculty of Nursing, King Abdulaziz University, Jeddah, 21589, Saudi Arabia, kau.edu.sa; ^2^ Faculty of Nursing, King Abdulaziz University, Jeddah, 21589, Saudi Arabia, kau.edu.sa; ^3^ Psychiatric and Mental Health Nursing Department, Faculty of Nursing, King Abdulaziz University, Jeddah, 21589, Saudi Arabia, kau.edu.sa

**Keywords:** epilepsy, quality of life, seizure

## Abstract

**Background:**

Epilepsy is one of the most common neurological conditions which affects 50 million individuals worldwide and has a high prevalence rate in Saudi Arabia (0.654%). Epilepsy negatively impacts the global health and lowers the quality of life (QoL) of affected individuals. Patients with epilepsy not only deal with the symptoms of the disease but also with the adverse effects of treatment and the worry of an unexpected crisis.

**Objectives:**

This study aimed to assess the QoL in patients with epilepsy in Jeddah, Saudi Arabia.

**Methodology:**

A descriptive cross‐sectional study was conducted at a governmental hospital on 101 patients who met the inclusion criteria. The QoL of patients with epilepsy was assessed using an Arabic version of the Quality of Life in Epilepsy Inventory 31 (QOLIE‐31) questionnaire to examine the QoL. Data analysis of the study was carried out by using descriptive statistics (SPSS) version 23.

**Results:**

The overall score was 51.2, indicating a decline in the QoL of patients with epilepsy. This score was compared with a published global normative mean QOLIE‐31 score of 59.8. The difference between the study mean and the global mean was statistically significant (one‐sample *t*‐test, t = −5.223, *p* < 0.001). The domains with the highest rankings were in the following order: overall QoL (61.9), energy/fatigue (52.3), cognitive function (51.9), social function (51.8), medication effects (45.9), emotional well‐being (45.7), and seizure‐worry (39.1).

**Conclusion:**

Epilepsy was associated with a reduced QoL among patients in Jeddah, particularly in the domains of seizure worry, emotional well‐being, and medication effects. These findings highlight the need for comprehensive management strategies that address not only seizure control but also the psychosocial aspects of epilepsy care.

## 1. Introduction

Epilepsy is one of the most common and disabling neurological conditions. Approximately 50 million people suffer from epilepsy worldwide (WHO, 2022). It is often diagnosed after a detailed medical history compilation, which is supported by electroencephalogram and imaging findings [1]. Epilepsy is a brain illness marked by an enduring persistent proclivity to generate epileptic seizures that are unprovoked by any immediate central nervous system damage, as well as the neurobiological, cognitive, psychological, and social implications of this condition [2].

The quality of life (QoL) of epileptic patients can drastically diminish due to the disorder itself, as well as due to any associated comorbid mood and psychiatric disorders, cognitive deficits, or pharmacological side effects [3]. QoL has been defined as “a comprehensive notion that comprises emotional, physical, material, and social well‐being of an individual” [4].

Several studies have evaluated the impact of epilepsy on QoL. A study on epilepsy and QoL found that epilepsy‐related characteristics, such as seizure occurrence and antiepileptic medicines, were negatively associated with psychosocial elements, such as depression symptoms, psychological dysregulation, and perceptual discrimination [5]. Conversely, Akosile et al. [6] reported that adults with epilepsy had a lower QoL and larger fatigue impact compared to apparently healthy people and that the severity of their seizures was higher than the general population. Thus, factors like seizure severity, exhaustion impact, and degree of weariness all negatively affect the QoL.

Several studies have also documented the impact of epilepsy on the older population. Baranowski [7] performed a systematic review to examine the implications of the disease on QoL of older epilepsy patients.

The research on patients with epilepsy indicates that several factors are associated with a lower QoL. These include the presence of psychiatric comorbidities, higher seizure frequency, the type and severity of seizures, longer duration of seizures, the need for multiple antiepileptic medications, gender, and a lower socioeconomic status [3–5].

The Quality of Life in Epilepsy Inventory‐31 (QOLIE‐31) tool was to establish the categories and recurrence of seizure‐related harm and their implications on overall life quality. It was reported that patients with epilepsy were more likely to sustain seizure‐related injuries, which can be serious but have little influence on their QoL.

Studies have also shown a correlation between knowledge of epilepsy and outlook on life [8]. Stigmatization, psychological problems, and lower QoL were found to be linked to a lack of understanding and preconceived notions about the condition. Yogarajah and Mula [9] investigated social cognition, mental comorbidities, and QoL of adults with epilepsy. They reported that epilepsy was associated with impairments in social cognition, which in turn related to difficulties in social functioning and well‐being.

Billakota et al. (2019) reported that more than one‐third of epilepsy patients experience relapses and an even higher proportion experience treatment‐related side effects [10]. Other studies reported that factors like monthly income, housing situation, antiepileptic medication side effects, anxiety comorbidity, perceived stigma, and seizure frequency had a significant effect on the health‐related QoL of patients with epilepsy [11]. Another study found that the QoL of epileptic patients from lower socioeconomic groups varied due to socioeconomic factors (Sadr et al., 2016) [12].

Epilepsy has a wide range of consequences that go beyond its direct impact, including a variety of difficulties that can lead to disability and a significantly impaired QoL (Vaurio et al., 2017) [13]. Previous studies have reported that epilepsy symptoms make a major difference in the happiness of people with epilepsy [14]. Approximately half of the patients with epilepsy do not have a particularly high health‐related QoL [15]. When compared to other neurological conditions, epilepsy has the highest disability‐adjusted life year ratios and significantly affects both sexes [16]. Despite there is a significant prevalence of epilepsy in Saudi Arabia (0.654%) (MOH, 2020), much is not known about how epilepsy affects QoL.

Although several international studies have investigated the effects of epilepsy on QoL, only a limited number of studies in Saudi Arabia have assessed the prevalence, clinical characteristics, and QoL of patients with epilepsy [17]. The unique cultural, social, and healthcare environments in Saudi Arabia may shape patients’ experiences in ways that differ from other populations, underscoring the necessity for data that is relevant to the local context. Consequently, this study seeks to assess the QoL among epilepsy patients in Jeddah, Saudi Arabia, aiming to fill the existing knowledge gap and provide evidence that can inform culturally sensitive patient care and interventions.

## 2. Materials and Methods

### 2.1. Study Design and Setting

This was a descriptive cross‐sectional study carried out on patients with epilepsy from Jeddah, Saudi Arabia, between March 2022 and April 2022. Participants were recruited using a combined approach: from outpatient and inpatient units of a governmental hospital, and via an online questionnaire distributed through social media platforms (WhatsApp and Twitter) with support from the Saudi Epilepsy Society. The governmental hospital was chosen due to the researcher’s access to administrative records and because it is the first hospital in Saudi Arabia dedicated to medical education.

### 2.2. Sampling and Sample Size

A total of 101 patients were recruited using convenience sampling. Raosoft software was used to determine the optimal sample size. Inclusion criteria were: a confirmed diagnosis of epilepsy, age ≥ 18 years, and fluency in Arabic or English. Patients were excluded if they did not have a physician‐confirmed diagnosis, were younger than 18 years, or had cognitive or speech difficulties preventing comprehension of the questionnaire.

### 2.3. Study Tool

The QOLIE‐31, developed by Rand in 1993 [18] and translated into Arabic by Alaa Eldin Elsharkawy (2015) [19], was used to assess patients’ QoL. Permission to use the Arabic version was obtained via email on February 27, 2022. The QOLIE‐31 consists of seven standardized subscales that assess different aspects of QoL in patients with epilepsy. The Seizure Worry subscale, which includes five items, evaluates concerns about the frequency and impact of seizures. The Emotional Well‐Being subscale, also comprising five items, assesses feelings of depression, anxiety, and overall mood related to epilepsy. The Energy/Fatigue subscale, with four items, measures perceived levels of energy and fatigue and how they affect daily life. Cognitive Functioning, consisting of six items, examines attention, memory, and concentration. The Medication Effects subscale, including three items, evaluates the impact of antiepileptic medications on daily functioning. Social Functioning, with five items, measures participation in social roles and activities. Finally, the Overall Quality of Life subscale, consisting of two items, captures the global perception of health and life satisfaction.

Each subscale is scored on a 0–100 scale, with higher scores indicating better QoL. The total QOLIE‐31 score is calculated as the weighted mean of the individual subscale scores.

While reliability testing (e.g., Cronbach’s alpha) was not conducted in this sample, previous studies have reported acceptable reliability for the Arabic QOLIE‐31 translation, with a final overall QOLIE‐31 score of 0.885 [20].

### 2.4. Pilot Study

The questionnaire was tested on 10% of the participants to investigate their ability to complete the questionnaire, assess the questionnaire’s clarity, and determine the need to add or delete certain items.

### 2.5. Data Collection Process

The data were collected by using Arabic version of the QOLIE‐31 developed by 1993, Rand [18], translated by Alaa Eldin Elsharkawy [19], which is a tool used to assess the QoL in epileptic patients. Hospital‐based participants completed the questionnaire during their visits, while the online version was distributed through social media with the assistance of the Saudi Epilepsy Society. All participants provided informed consent prior to participation.

### 2.6. Statistical Analysis

Means, percentages, frequencies, and standard deviations were used to characterize the data in this study. Data were analyzed using descriptive (SPSS) software version 23. A one‐sample *t*‐test was performed to compare the study’s mean QOLIE‐31 score with the global normative mean. Independent samples *t*‐tests were used to examine differences in QOLIE‐31 scores according to sex, nationality, and occupational status. One‐way analysis of variance (ANOVA) was conducted to examine differences in QOLIE‐31 scores across age, educational level, and marital status groups.

### 2.7. Ethical Considerations

The study design was approved by the Nursing Research Ethics Committee of the Faculty of Nursing (NREC Serial no: Ref no 2B.40) on February 24, 2022 and the Unit of Biomedical Ethics at the governmental hospital on March 22, 2022 NCBE Registration No: (HA‐02‐J‐008) (Reference No 158‐22). All study procedures were performed in accordance with the rules and regulations set forth in the Declaration of Helsinki (DoH‐Oct2008). Participants provided written informed consent, and data were stored confidentially on a password‐protected laptop accessible only to the research team.

## 3. Results

### 3.1. Demographic Data

Analysis of the participant’s demographic details showed that 48.5% were aged 18–22 years, 28.7% were 23–26 years, 8.9% were 27–30 years, and 13.9% were above 30 years. The study population comprised 21.8% males and 78.2% females. Of the study population, 2% had an elementary school education, 3% middle school education, 29.7% high school education, and 65.3% had a bachelor’s degree. Of all the participants, 80.2% were single, 13.9% were married, and 5.9% were divorced. Only 23.8% of the participants were employed, while the remaining 76.2% were unemployed. Majority (85.1%) of the participants were Saudi.

### 3.2. QoL

The data of seizure worry domain showed that the final mean score of the seizure worry domain was 39.1. The fear of having a seizure within the next few months ranked the highest (46.5), followed by the fear of embarrassment or other social problems resulting from having a seizure within the next few months (43.6), fear of hurting oneself during a seizure (40.1), botheration of seizures (36.4), and worry of having another seizure (29.3).

Regarding the overall QOL domain, the final score of the overall QOL domain was 61.9. Among the items, QoL during the past 4 weeks (64.6) scored the highest, followed by the overall rate of QoL (59.1).

In relation to the emotional well‐being, the final score of this domain was 45.7. Among the items, “have you been a happy person” scored the highest (55.84), followed by “have you felt calm and peaceful” (53.07), “have you felt so down in the dumps that nothing could cheer you up” (46.93), “have you felt downhearted and blue” (40.59), and “have you been a nervous person” (31.88).

Data on energy and fatigue domain showed that the domain’s final score was 52.03, with the items listed in the following order: “Did you feel full of pep” (63.76), “Did you have a lot of energy” (58.42), “Did you feel worn out” (44.55), and “Did you feel exhausted” (41.39).

However, regarding cognitive function, data analysis showed that 51.9 was the final score of this domain. The item “Have you had any trouble with your memory in the past four weeks?” scored 59.1. The next three highest scores were for “Difficulty concentrating on reading” (55.5), “Difficulty concentrating on doing one thing at a time” (51.9), and “Difficulty reasoning and solving problems” (50.5). The parameters “Did you have trouble remembering what someone told you” (49.9) and “Memory difficulties” (45.0) scored the least.

The ultimate score for the medication’s effect domain was 45.9. Among the items, antiepileptic drug side effects on the brain scored the highest (48.8), followed by effects on the body (48.3). The parameter “How concerned are you that the long‐term effects of the medicines you take could be harmful?” (40.6) scored the least.

The total score for the social functioning domain was 51.8, and the item “has your health prevented you from engaging in social activities (such as visiting friends or close relatives)?” scored the highest (58.2), followed by concerns about driving (53.7), leisure time (52.7), social limitations (51.2), and work limitations (43.6).

The overall score of the various domains of the QOLIE‐31 was 51.2 (Table 1). Among these, “Overall QoL” scored the highest (61.9), followed by energy/fatigue (52.3), cognitive functioning (51.9), social functioning (51.8), medication effects (45.9), emotional well‐being (45.7), and seizure‐worry (39.1).

**TABLE 1:** Calculation of QOLIE‐31 overall score.

QOLIE‐31	Final score	Weight	Subtotal score
Seizure‐worry	39.1	0.08	3.1
Overall QoL	61.9	0.14	8.7
Emotional well‐being	45.7	0.15	6.9
Energy/fatigue	52.3	0.12	6.3
Cognitive functioning	51.9	0.27	14.0
Medication effects	45.9	0.03	1.4
Social functioning	51.8	0.21	10.9
Overall score	51.2		

Table 2 shows the differences in the QOLIE‐31 domains in terms of sex, nationality, and occupational status. No significant differences were observed in the QOLIE‐31 domains according to sex, nationality, and occupational status (all *p*‐values > 0.05).

**TABLE 2:** Difference in domains of the QOLIE‐31 according to sex, nationality, and occupational status.

QOLIE‐31	Factor	Test	Statistics	*p*‐value
Seizure‐worry	Sex	Independent samples test	0.137	0.891
	Nationality	Independent samples test	1.451	0.15
	Occupational status	Independent samples test	−0.453	0.652

Overall QoL [1]	Sex	Independent samples test	1.038	0.302
	Nationality	Independent samples test	0.207	0.836
	Occupational status	Independent samples test	−0.828	0.41

Emotional well‐being	Sex	Independent samples test	1.058	0.292
	Nationality	Independent samples test	0.656	0.513
	Occupational status	Independent samples test	−0.941	0.349

Energy/fatigue	Sex	Independent samples test	0.563	0.575
	Nationality	Independent samples test	1.106	0.272
	Occupational status	Independent samples test	−1.298	0.197

Cognitive functioning	Sex	Independent samples test	0.157	0.876
	Nationality	Independent samples test	1.531	0.129
	Occupational status	Independent samples test	0.299	0.765

Medication effects	Sex	Independent samples test	−0.793	0.429
	Nationality	Independent samples test	0.632	0.529
	Occupational status	Independent samples test	−1.121	0.265

Social functioning	Sex	Independent samples test	−0.139	0.889
	Nationality	Independent samples test	0.253	0.801
	Occupational status	Independent samples test	−1.903	0.06

Overall score	Sex	Independent samples test	0.288	0.774
	Nationality	Independent samples test	1.262	0.21
	Occupational status	Independent samples test	−1.051	0.296

Table 3 shows the differences in the QOLIE‐31 domains according to age, educational level, and marital status. No significant differences were observed in the QOLIE‐31 domains according to age and educational level (*p*‐values > 0.05). Conversely, significant differences were observed in the context of marital status in the overall QoL (F = 4.092, *p*‐value = 0.02), emotional well‐being (F = 4.986, *p* = 0.009), energy/fatigue (F = 3.377, *p*‐value = 0.038), and cognitive functioning domain (F = 3.205, *p*‐value = 0.045). However, no significant differences were noted in the medication effects, social functioning, and overall score domain (*p*‐value > 0.05).

**TABLE 3:** Difference in domains of the QOLIE‐31 according to age, educational level, and marital status.

QOLIE‐31	Factor	Test	Statistics	*p* value
Seizure‐worry	Age	One‐way ANOVA [2]	0.669	0.573
	Educational level	One‐way ANOVA	0.740	0.530
	Marital status	One‐way ANOVA	0.569	0.568

Overall QOL [3]	Age	One‐way ANOVA	0.636	0.594
	Educational level	One‐way ANOVA	0.779	0.508
	Marital status	One‐way ANOVA	4.092	0.02

Emotional well‐being	Age	One‐way ANOVA	0.883	0.453
	Educational level	One‐way ANOVA	0.634	0.595
	Marital status	One‐way ANOVA	4.986	0.009

Energy/fatigue	Age	One‐way ANOVA	0.773	0.512
	Educational level	One‐way ANOVA	0.373	0.773
	Marital status	One‐way ANOVA	3.377	0.038

Cognitive functioning	Age	One‐way ANOVA	1.536	0.210
	Educational level	One‐way ANOVA	0.210	0.889
	Marital status	One‐way ANOVA	3.205	0.045

Medication effects	Age	One‐way ANOVA	0.669	0.573
	Educational level	One‐way ANOVA	1.638	0.186
	Marital status	One‐way ANOVA	0.078	0.925

Social functioning	Age	One‐way ANOVA	1.789	0.154
	Educational level	One‐way ANOVA	0.176	0.913
	Marital status	One‐way ANOVA	0.852	0.430

Overall score	Age	One‐way ANOVA	0.779	0.509
	Educational level	One‐way ANOVA	0.345	0.793

The results showed that the overall score was 51.2, indicating a decline in the QoL of patients with epilepsy. This score was compared with a published global normative mean QOLIE‐31 score of 59.8. The difference between the study mean and the global mean was statistically significant (one‐sample *t*‐test, t = −5.223, *p* < 0.001). The domains with the highest rankings were in the following order: overall QoL (61.9), energy/fatigue (52.3), cognitive function (51.9), social function (51.8), medication effects (45.9), emotional well‐being (45.7), and seizure‐worry (39.1).

## 4. Discussion

The global mean QOLIE‐31 score in this study was 59.8, indicating that patients with epilepsy experienced a significant decline in QoL. A previous study also reported an overall score of 51.2, indicating decreased QoL [21].

Fiest et al. [15] found higher incidence and prevalence of epilepsy in the youngest and oldest age groups. However, in our study, age did not play a significant role in determining the QoL.

Patients with epilepsy and adequate education are more likely to comply with the medications because they understand the illness process, seizure triggers, and appropriate seizure management. Therefore, the standard of living for this demographic group is more likely to improve. In our study, educational level did not have a significant effect on the QoL after adjusting for confounding variables. These findings were in concordance with a previous study on epilepsy patients in Mexico (Camara‐Lemarroy et al., 2017) [22].

Majority of our study participants were unmarried (80.2%), whereas 13.9% were married and 5.90% were divorced. The common belief regarding the hereditary nature of epilepsy can explain why so many people with the disorder choose not to start their families. Similar results were reported in Sudan by Ibrahim (2020), where 57.3% of the participants were single and 2.9% were divorced [23].

An Iranian study reported the highest domain score for energy/fatigue [16], while several studies found it to have the lowest score on the QOLIE‐31 questionnaire [21–24]. The effect of medications was rated highest in a study conducted in Northern Ethiopia [25]. Factors such as lack of restorative sleep after a seizure [26] and pharmaceutical side effects were reported to contribute to fatigue [27]. Anxiety about having an epileptic seizure received the lowest score in all domains. Another study conducted in Qassim by Altwijri et al. (2021) found that employment status was substantially related to both the total score and each of the seven subscales [17]. We found that the overall QoL score and each of the seven subscale scores varied significantly according to marital status. Australian researchers found no correlation between demographic variables and QoL measures across all seven scales [17].

In this study, a marginal effect of emotional well‐being on QoL was only (45.7%). While the number of episodes was highly related to emotional well‐being and cognition subscales in another study, the findings revealed that a low QoL score was associated with low mood [24].

The QoL of patients with epilepsy was found to be unaffected by the drug effect in our study. Contrarily, Abadiga et al. [11] reported that the adverse medication effects reduced patients’ QoL in a study conducted in Ethiopia.

Our study showed that epilepsy patients struggled in social situations. Similarly, a Chinese study reported that patients with epilepsy often experienced social isolation, which was strongly correlated with poor health‐related QoL [20].

Mrabet et al. (2004) along with Shakir and Al‐Asadi (2012) reported no association between marital status and QoL in Tunisian [28] and Iraqi [29] patients, respectively. The results from the Iraqi and Tunisian samples did not seem to affect the employment rates and were in concordance with our results. Age and education level were found to have substantial effects on participants’ overall satisfaction in Iraq.

### 4.1. Limitations of the Study

Although our sample was collected from the community of Jeddah in Saudi Arabia at a governmental hospital in Jeddah, the sample size was insufficient to generalize the results. In addition, while several sociodemographic factors were explored using one‐way ANOVA, we recognize that multiple variables may interact to influence QoL. Therefore, multivariate analysis should be considered in future studies to account for potential confounders. Moreover, the study focused primarily on sociodemographic variables and QOLIE‐31 outcomes and did not assess clinical epilepsy characteristics such as age of onset, seizure frequency, type of seizures, or specific antiepileptic drug usage, which may significantly impact QoL and should be considered in future research.

### 4.2. Implications for Nursing Practice

Nurses are essential in evaluating and supporting the QoL of patients suffering from epilepsy. Regular assessments utilizing validated instruments like the QOLIE‐31 can assist in identifying patients who are at risk of experiencing poor QoL and in developing personalized care strategies. It is crucial for nurses to educate patients about seizure management, adherence to medication, and lifestyle changes aimed at reducing seizure occurrences and improving overall well‐being. Furthermore, nurses can provide psychosocial support to tackle emotional difficulties, diminish stigma, and enhance social interactions. Incorporating these approaches into standard nursing care can lead to improved patient outcomes and contribute to a better QoL for those living with epilepsy.

### 4.3. Implications for Future Research

We suggest creating a training program for health care providers regarding patients with epilepsy and their QoL. Moreover, adequate measures should be taken to increase the awareness regarding epilepsy in different settings (universities, schools, other healthcare centers, and hospitals).

More research is needed to find ways to improve the lives of people with epilepsy. We also recommend that future researches should investigate the impact of epilepsy on QoL by expanding the sample size to include a greater variety of patients. We also suggest utilizing a broader range of data collection techniques, including self‐reporting questionnaires and interviews, and collecting data from multiple perspectives. Future studies should investigate the economic status of patients with epilepsy to determine how it affects their QoL, an aspect that was not included in our demographic survey.

## 5. Conclusion

Epilepsy has a significant impact on various dimensions of patients’ QoL, encompassing social interactions, cognitive abilities, energy levels, and emotional well‐being. Findings of this study underscore the necessity of holistic care that not only focuses on controlling seizures but also incorporates psychosocial support, patient education, and the management of medication‐related side effects. Nurses and healthcare professionals are crucial in evaluating QoL, delivering personalized interventions, and assisting patients in navigating the difficulties associated with epilepsy. Implementing these strategies can enhance overall well‐being, improve adherence to treatment, and facilitate culturally sensitive care for patients in Jeddah, Saudi Arabia.

## Author Contributions

Conceptualization: Ruba Alharazi, Yara Abushosha, Nouf A. Aldajany, Nouf J. Alreziq, and Lujain A. Sallam; methodology: Ruba Alharazi, Yara Abushosha, Nouf A. Aldajany, Nouf J. Alreziq, Lujain A. Sallam, Aisha Alhofaian, and Loujain S. Sharif.; software: Yara Abushosha, Nouf A. Aldajany, and Nouf J. Alreziq; validation: Ruba Alharazi, Yara Abushosha, Nouf A. Aldajany, Nouf J. Alreziq, and Lujain A. Sallam; formal analysis: Ruba Alharazi, Yara Abushosha., Nouf A. Aldajany, Nouf J. Alreziq, Lujain A. Sallam, Aisha Alhofaian, and Loujain S. Sharif.; investigation: Ruba Alharazi, Yara Abushosha, Nouf A. Aldajany, Nouf J. Alreziq, and Lujain A. Sallam; resources: Yara Abushosha, Nouf A. Aldajany, Nouf J. Alreziq; data curation: Ruba Alharazi, Yara Abushosha, Nouf A. Aldajany, Nouf J. Alreziq, Lujain A. Sallam, Aisha Alhofaian, and Loujain S. Sharif.; writing–original draft preparation: Yara Abushosha, Nouf A. Aldajany, and Nouf J. Alreziq; writing–review and editing: Ruba Alharazi, Yara Abushosha, Nouf A. Aldajany, Nouf J. Alreziq, Lujain A. Sallam, Aisha Alhofaian, and Loujain S. Sharif.; visualization: Ruba Alharazi, Yara Abushosha, Nouf A. Aldajany, Nouf J. Alreziq, and Lujain A. Sallam; supervision: Ruba Alharazi and Loujain S. Sharif.; project administration: Ruba Alharazi, Yara Abushosha, Nouf A. Aldajany, Nouf J. Alreziq, and Lujain A. Sallam.

## Funding

The project was funded by KAU Endowment (WAQF) at King Abdulaziz University, Jeddah, Saudi Arabia. The authors, therefore, acknowledge with thanks WAQF and the Deanship of Scientific Research (DSR) for technical and financial support.

## Disclosure

This manuscript was drafted in accordance with the STROBE Statement for cross‐sectional studies. All authors have read and agreed to the published version of the manuscript.

## Ethics Statement

This study was conducted in accordance with the Declaration of Helsinki and approved by the Nursing Research Ethics Committee of Faculty of Nursing, Saudi Arabia and the Institutional Review Board of King Abdulaziz University Hospital in Jeddah, Saudi Arabia.

## Consent

Informed consent was obtained from all subjects involved in this study.

## Conflicts of Interest

The authors declare no conflicts of interest.

## Data Availability

Data are available upon reasonable request from the corresponding author.
